# Accuracy of virtual planning in orthognathic surgery: a systematic review

**DOI:** 10.1186/s13005-020-00250-2

**Published:** 2020-12-04

**Authors:** Ali Alkhayer, József Piffkó, Carsten Lippold, Emil Segatto

**Affiliations:** 1 Craniofacial Unit, Department of Oral & Maxillofacial Surgery, University of Szeged, Tisza Lajos krt. 97, Szeged, Hungary; 2 Department of Oral & Maxillofacial Surgery, Faculty of Medicine, University of Szeged, Kálvária sugárút. 57, Szeged, Hungary; 3 Department of Orthodontics, Universitätsklinikum Münster, Albert-Schweitzer-Campus 1, Gebäude W30, Waldeyerstraße 30, 48149 Münster, Germany

**Keywords:** Surgery, computer-assisted, Orthognathic surgery, Dentofacial deformities, Cone-beam computed tomography

## Abstract

**Background:**

The elaboration of a precise pre-surgical plan is essential during surgical treatment of dentofacial deformities. The aim of this study was to evaluate the accuracy of computer-aided simulation compared with the actual surgical outcome, following orthognathic surgery reported in clinical trials.

**Methods:**

Our search was performed in PubMed, EMBASE, Cochrane Library and SciELO for articles published in the last decade. A total of 392 articles identified were assessed independently and in a blinded manner using eligibility criteria, out of which only twelve articles were selected for inclusion in our research. Data were presented using intra-class correlation coefficient, and linear and angular differences in three planes.

**Results:**

The comparison of the accuracy analyses of the examined method has shown an average translation (< 2 mm) in the maxilla and also in the mandible (in three planes). The accuracy values for pitch, yaw, and roll (°) were (< 2.75, < 1.7 and < 1.1) for the maxilla, respectively, and (< 2.75, < 1.8, < 1.1) for the mandible. Cone-beam computed tomography (CBCT) with intra-oral scans of the dental casts is the most used imaging protocols for virtual orthognathic planning. Furthermore, calculation of the linear and angular differences between the virtual plan and postoperative outcomes was the most frequented method used for accuracy assessment (10 out of 12 studies) and a difference less than 2 mm/° was considered acceptable and accurate.

When comparing this technique with the classical planning, virtual planning appears to be more accurate, especially in terms of frontal symmetry.

**Conclusion:**

Virtual planning seems to be an accurate and reproducible method for orthognathic treatment planning. However, more clinical trials are needed to clearly determine the accuracy and validation of the virtual planning in orthognathic surgery.

**Supplementary Information:**

The online version contains supplementary material available at 10.1186/s13005-020-00250-2.

## Background

Two-dimensional (2D) radiographs and manual model surgery are essential parts of the preoperative planning for orthognathic surgery. However, this approach has its limitations, especially in the case of patients with major facial deformity or asymmetry [1], as 2D cephalometric images cannot provide full information about the 3D structures.

When conventional 2D surgical plans are executed, unexpected problems, such as a bony collision in the ramus area, the discrepancy in pitch, roll and yaw rotation, midline difference and chin inadequacy may occur [2].

When two-jaw surgery is performed, an inter-occlusal splint is fabricated to work as an intermediate guide for repositioning the maxilla relative to the intact mandible [3]. Any variation between the plan and the plaster model surgery could lead to a poorly fabricated wafer, which in turn could lead to unexpected (and often undesirable) results, regardless of how skillfully and carefully the surgery is performed [3].

These examples illustrate that the elaboration of a precise pre-surgical plan is of utmost importance when it comes to correcting dentofacial deformities.

Computer-aided surgical simulations using cone beam computed tomography (CBCT) images have revolutionized orthodontics and have been adapted for orthognathic surgery (OGS) to facilitate cephalometric analysis, surgical simulation and splint fabrication [4–9].

In particular, the visualization of skeletal complexities within an asymmetric dentofacial deformity has been greatly enhanced through three dimensional (3D) modeling, which can demonstrate the extent of yaw rotation in the maxilla and mandible, occlusal plane canting and differential length of a mandibular body or the ramus [1, 10, 11]. The 3D simulation method has been accepted for planning in orthognathic surgery and led to significant improvements in surgical outcomes [1, 9, 12]. Intraoperative efficiency has also improved with the fabrication of the templates and jigs to reproduce gaps or spacing between the osteotomies depicted in the virtual plan. These jigs may reinforce intraoperative accuracy of the clinical movement of the virtual plan and aid in orienting and positioning bony segments [10, 13–18]. Thus, the aim of this systematic review is to assess the accuracy of computer-aided planning in orthognathic surgery.

## Methods

A systematic search was conducted of electronic and printed articles that have been published in the period (2007–2017) on virtual planning for orthognathic surgery and in the English language. The databases used were PubMed, EMBASE, Cochrane Library and SciELO. Keywords and Boolean operators (‘OR’ and ‘AND’) were used to join the terms related to orthognathic surgery and virtual planning.

### Search strategy

#### Main search

The systematic search was done by one of the authors (A.A.). The search of PubMed was conducted using the following medical subject heading (MeSH) terms: [(‘Orthognathic Surgery’ OR ‘Surgery, Orthognathic’ OR ‘Maxillofacial Orthognathic Surgery’ OR ‘Orthognathic Surgeries, Maxillofacial’ OR ‘Orthognathic Surgery, Maxillofacial’ OR ‘Surgery, Maxillofacial Orthognathic’ OR ‘Orthognathic Surgical Procedures’ OR ‘Procedure, Orthognathic Surgical’ OR ‘Surgical Procedure, Orthognathic’ AND (‘Surgery, virtual planning’ OR ‘virtual planning Surgery’ OR ‘Computer Assisted Surgery’ OR ‘virtual planning ,Surgery’ OR ‘Surgery, virtual planning’ OR ‘virtual planning Design’ OR ‘virtual planning Designs’ OR ‘Design, virtual planning’ OR ‘virtual planning Manufacturing’ OR ‘Manufacturing, virtual planning’)].

The same search strategy was applied to the Cochrane Library since this also uses MeSH terms.

For the search of EMBASE, the entry terms ‘orthognathic surgery’ AND ‘virtual planning surgery’ were used to carry out a specific search.

Health sciences descriptors were used to search the SciELO databases, ‘orthognathic surgery’ AND ‘virtual planning’ were performed.

#### Eligibility of the studies

The eligibility of the studies was determined by the author (A.A.), observing the following criteria: (1) the main theme of the paper had to focus on virtual planning for orthognathic surgery; (2) the study had to be original and interventional; (3) the surgical procedure had to be virtually planned with a virtual surgical splint; (4) accuracy measures had to be presented for the surgical procedure; (5) the sample size of the trial had to be ≥10. The latter criterion was determined somewhat arbitrarily, as a reasonable minimum, given the small sample sizes of these studies in general.

### Main search

**Fig. 1:**
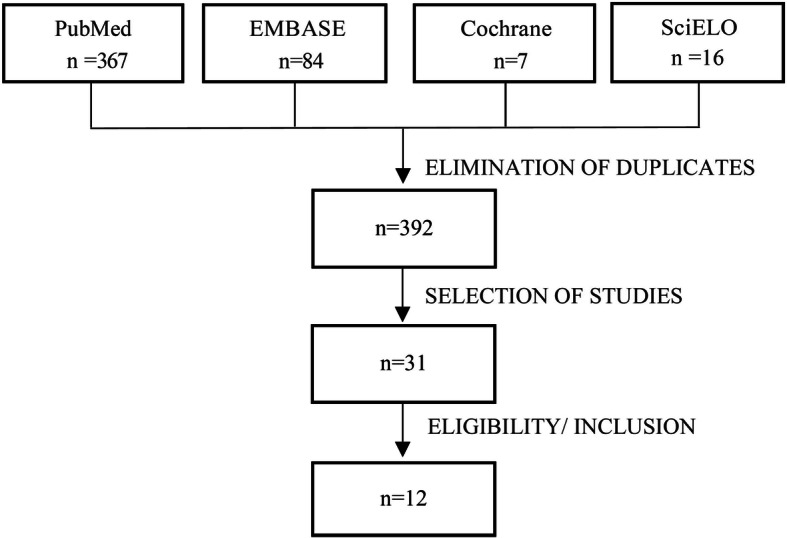
Flowchart of the review process

Three hundred and sixty-seven articles were found in PubMed, 84 in EMBASE, 7 in Cochrane Library and 16 in SciELO. Duplicate papers were removed, leaving a total of 392 possible studies, that have been read and 31 of these were chosen for full-text reading (Fig. 1).

### Eligibility assessment

As part of the eligibility assessment, 31 studies were read in full. At the end of this analysis, only twelve papers were included in the sample for our systematic review. The other 19 studies were excluded for the following reasons: virtual surgical planning for orthognathic surgery was not the main focus of the paper [19],the paper was not an intervention study [17], or it was not original [5, 20, 21], the surgical procedure did not involve a computer-assisted virtual surgical splint [22–24], the accuracy measurements for the surgical procedure were not provided [25–29] and the sample size was less than 10 [16, 22, 30–33].

### Quality assessment of the included articles

The quality of the papers was assessed using an adaptation of the bias analysis proposed by Clementini and colleagues [34]. The criteria were the presence or absence of the following: sample randomization, blind assessment, statistical analysis, defined inclusion and exclusion criteria and reporting of follow-up. With respect to the risk of bias for each analyzed study, papers containing all the above items were considered low risk, studies lacking one or two items were missing were deemed medium risk, and investigations that lacked three or more items were considered high risk.

## Results

Descriptive data of the included studies (sample size, age, gender and type of facial deformity) are presented in (Table 1).

**Table 1:** Descriptive data of the included studies

Authors, year and country of origin	Type of study	Sample size	Age: mean, SD (variation)	Gender	Type of facial deformity
**De Rio et al. 2017 Italy** [35]	Retrospective observational study	N: 49 patients	Mean: 26.4 years	19 males 30 females	Angle class II: 16 Angle class III: 20 Open bite: 4 Facial asymmetry: 9
**Ritto et al. 2017 Brazil** [36]	Retrospective study	N: 30 patients: CMS group: 15 VSP group: 15	NA	CMS group: 8 females 7 males VSP group: 5 females 10 males	CMS group: 4 skeletal class II malocclusion 11 skeletal class III malocclusion VSP group: 1 skeletal class I malocclusion, 2 presented class II malocclusion, 12 presented class III malocclusion
**Ho et al. 2017 Taiwan** [1]	Prospective case series, A	N: 30 patients	Mean: 22.4 years Range: (18–26 years)	22 females 8 males	Class III malocclusion and facial asymmetry
**Chin et al. 2017 Germany** [37]	A comparative study	N: 10 patients	Mean: 25.3 years Range: (18–41) years	4 males 6 females	8 Class III, Prognathism of Mandible 2 Class II retrognathism of Mandible
**Stokbro et al. 2016 USA** [38]	A comparative retrospective study	N: 30 patients CMS group: 15 VSP group: 15	Mean: 23.1 ± 6.8 years Median: 21 years Range: (18–42) years	10 males 20 females	NA
**Baan et al. 2016 Netherlands** [39]	Prospective study	N: 10 patients	Mean: 26.5 years Range: (17–45) years	4 Males 6 Females	Skeletal Class II profile
**Zhang et al. 2016 China** [40]	A comparative retrospective study	N: 30 patients	Range: (19–30) years	16 males 14 females	(*n* = 27) Skeletal class III profile, retrognathia of upper jaw, Prognathia of lower jaw . (*n* = 3) Skeletal class II profile prognathia of upper jaw Retrognathia of lower jaw.
**De Rio et al. 2014 Italy** [41]	Randomized controlled clinical trial	N: 20 patients Virtual splint: 10 Classic splint: 10	Virtual splint: Range: (21–54) years Classic splint: Range: (24–47) years	Overall: 10 M, 10 F Virtual splint: 3 M, 7 F Classic splint: 7 M, 3 F	Class II/class III: NA All asymmetrical
**Hsu et al. 2013 USA** [6]	A Prospective Multicenter Study	N: 65 patients Houston: 41 Portland: 11 New York: 13	Houston: mean 25 range: (15–51) Portland: mean 26.7 range (15–51) NewYork: mean 26.7 range (16–46)	Houston 23 M, 18 F Portland: 3 M, 8 F New York: 5 M, 8 F	NA
**Sun et al. 2013 Belgium** [7]	Prospective case series	N: 15 patients	NA	NA	NA
**Zinser et al. 2013 Germany** [42]	Non-randomized clinical trial	N: 28 patients Virtual splint: 8 Classic splint: 10 Surgical navigation: 10	Overall: 20.8 ± 4.9 (18–35) years Virtual splint: 21.6 ± 5.45 (19–35) Classic splint: 20.6 ± 2.6 (18–26) Surgical navigation:20.5 ± 4.1(18–32)	Overall: 15 M, 13 F Virtual splint: 4 M, 4 F Classic splint: 6 M, 4 F Surgical navigation:5 M,5 F	Overall: 5 class II, 23 class III Virtual splint: 8 class III Classic splint: 4 class II, 6 class III Surgical navigation: 1 class II, 9 class III
**Centenero and Hernández-Alfaro**. **2012, Spain** [43]	Prospective case series	N: 16 patients	NA	NA	9 class II 7 class III

*SD* standard deviation, *NA* no information provided by the authors, *CMS* conventional model surgery, *VSP* vitual surgical planning, *M* male, *F* female

The imaging protocols and the software used for surgical planning varied substantially among the studies, These variations are shown in (Table 2).

**Table 2:** Imaging protocols and software used in the incuded studies

Author and year	Imaging method	Postoperative period of scanning the dentofacial complex	Imaging of dental arches	Software used for virtual planning
**De Rio et al. 2017** **Italy** [35]	CBCT	3rd–5th postoperative days	NA	(Maxilim®, Medicim, Nobel Biocare Group, Mechelen, Belgium). (Dolphin®, Dolphin Imaging and Management Solutions, Chatsworth, CA, USA) for Cephalometric analysis
**Ritto et al. 2017** **Brazil** [36]	CT	≥10 days after surgery	Scan of the plaster models using a 3D laser scanner	Dolphin Imaging software (Dolphin Imaging and Management Solutions, Chatsworth, CA, USA)
**Ho et al. 2017** **Taiwan** [1]	CBCT	1 month after surgery	NA	SimPlant (Materialize, Leuven,Belgium) Dolphin software (Dolphin Imaging and Management solutions, Chatsworth, California)
**Chin et al. 2017** **Germany** [37]	(CT)	1 month postoperatively	Scan of the plaster models under final occlusal position	Dolphin Imaging 11.8 Premium Assesmant tool / software: Geometric Studio® (Geomagic, Morrisville, NC, USA)
**Stokbro et al. 2016** **USA** [38]	CBCT	1 week after surgery	NA	Dolphin 3D (Dolphin Imaging and Management, Chatsworth, CA, USA)
**Baan et al. 2016** **Netherlands** [39]	CBCT	one to three weeks after surgery	CBCT triple scan procedure	Maxilim (Medicim NV, Mechelen, Belgium) Assessment tool/software: OrthoGnatic Analyzer
**Zhang et al. 2016** **China** [40]	(CT)	1 month postoperatively	surface scanning of the dental arch	Dolphin Imaging 11.7 Premium. Mimics software (version 10.01; Materialise, Leuven, Belgium
**De Rio et al. 2014** **Italy** [41]	CBCT	6 months	CBCT triple scan procedure	Maxilim (Medicim Nobel Biocare Group, Belgium) virtual planning and manufacturing of virtual splint
**Hsu et al. 2013** **USA** [6]	CT	6 weeks postoperatively	Scan of plaster models with reference points	Simplant OMS (Materialise Dental, Maryland, USA) Assesmant tool / software: 3DS max (Autodesk, CA, USA)
**Sun et al. 2013** **Belgium** [7]	CBCT	6 weeks	Scan of bite registration with reference points for image fusion with CT	Amira (Visage Imaging, Germany) virtual planning and manufacturing of virtual splint VisCAM (Marcam Engineering GmbH, Germany)
**Zinser et al. 2013** **Germany** [42]	CT CBCT	6 weeks	Scan of plaster models	SimPlant Pro OMS 10.1 (Materialise Dental, Belgium)
**Centenero and Hernández-Alfaro. 2012, Spain** [43]	CT CBCT	3 months	Scan of plaster models	SimPlant Pro OMS 10.1 (Materialise Dental, Belgium.

*CT* computed tomography, *CBCT* cone beam computed tomography, *3D* three dimensional, *NA* data not provided by the authors, *CBCT* cone beam computed tomography

The included studies also varied in the type of surgical plan and virtual splints, as well as in the method used for the assessment of accuracy. These variations we summarized in (Table 3).

**Table 3:** Variation in the type of surgical plan, virtual splints and the methodology of accuracy assessment in the included studies

Author, year and country of origin	Surgical planning	Surgical splint	Surgical splint
**De Rio et al. 2017 Italy** [35]	Bimaxillary surgery	Digital intermediate splints to guide osteotomies.	linear and angular differences to record the vector differences. Wilcoxon signed-rank test and the Mann-Whitney U test were used to analyze the differences between subgroups of the population
**Ritto et al. 2017 Brazil** [36]	Bimaxillary surgery	An intermediate splint was fabricated virtually	The mean linear difference between the planned movement and the movement obtained for each reference point was calculated, Intraclass correlation coefficient was used for the statistical analysis. The difference in precision between (2D,3D) methods was determined by t-test for independent samples.
**Ho et al. 2017 Taiwan** [1]	Bimaxillary surgery	Single occlusal splint	Linear and angular distance between reference points on the x (pitch), y roll), and z (yaw) planes
**Chin et al. 2017 Germany** [37]	9: bimaxillary surgery 1: repositioning of the lower jaw.	Two surgical splints: The first splint would guide the repositioning of segmented maxilla The second one is the final position of lower jaw.	linear and angular measurements were calculated and compared by using a paired t test
**Stokbro et al. 2016 USA** [38]	Bimaxillary surgery, Bimaxillary surgery with segmentation of the maxilla, Bimaxillary surgery with genioplasty, Bimaxillary surgery with segmentation of the maxilla and genioplasty	Surgical splints and surgical calipers.	The mean linear differences between the virtual plan and the postoperative outcomes were calculated and compared using Wilcoxon signed-rank test with 95% confidence intervals, Mann Whitney and U-test were used to analyze differences between the dependent groups. The clinical success criterion was set at a difference of less than 2 mm
**Baan et al. 2016 Netherlands** [39]	Bimaxillary surgery	Inter-occlusal wafer was milled based on the virtual planning.	Intraclass correlation coefficient (ICC) was calculated to evaluate the interobserver and intra-observer variability for the rotational and translational measurements of the maxilla and mandible.
**Zhang et al. 2016 China** [40]	LeFort I osteotomy of the maxilla combined with bilateral SSRO of the mandible. Genioplasty was performed, if indicated (17 patients)	Series of surgical templates: final occlusal splint, two pairs of 3D arms and a pair of bone attachments with indication of osteotomy line	Linear and angular differences between simulated and postoperative models were calculated and statically analyzed using Paired t test .
**De Rio et al. 2014 Italy** [41]	Clinical and 3D analysis Bimaxillary surgery (20), planning through maxilla (NA) and mandible (NA)	Occlusal splint	Linear and angular distance between the reference points and the reference lines in relation to FHP, CP, MFP, and the frontal process of the zygomatic bone 3D imaging (voxel-based)
**Hsu et al. 2013 USA** [6]	Bimaxillary surgery planning through maxilla	Occlusal splint, Bone splint (chin)	Calculating linear and angular differences, Bland and Altman’s statistical method
**Sun et al. 2013 Belgium** [7]	Bimaxillary surgery planning through maxilla	Occlusal splint	Linear and angular distance between reference points on the x (pitch), y (roll), and z (yaw) planes, 3D imaging (surface-best-fit),3ds Max (Autodesk Inc., USA)
**Zinser et al. 2013 Germany** [42]	Clinical and 3D analysis Bimaxillary surgery (28), planning through maxilla	Occlusal splint, Bone splint (maxilla and mandibular condyle)	Linear distance between the reference points for the x, y, and z planes in 3D imaging (voxel-based)
**Centenero and Hernández-Alfaro .2012, Spain** [43]	(15) Bimaxillary surgery, (1) Single maxillary surgery	Occlusal splint	Intra-class correlation coefficient (ICC) of the reference lines and angles; concordance level 3D imaging (NA)

*3D* three-dimensional, *NA* no information provided by the authors, *FHP* Frankfort horizontal plane, *CP* coronal plane, *MFP* midfacial plane, *N* nasion point

The actual accuracy values are presented in detail in Additional file 1 (Table S1).

Finally, the papers included in this review were assessed as being medium quality, since the risk of bias was considered medium in ten studies of the twelve. The risk of bias assessement for the included studies are presented in (Table 4).

**Table 4:** Risk of bias assessment of the included studies

Quality criteria for studies	Sample randomization	Blind assessment	Statistical analysis	Defined inclusion and exclusion criteria	Report of follow-up	Risk of bias assessment
**De Rio et al. 2017, Italy** [35]	No	No	Yes	Yes	Yes	Medium Risk
**Ritto et al. 2017, Brazil** [36]	No	No	Yes	Yes	Yes	Medium Risk
**Ho et al. 2017, Taiwan** [1]	No	No	Yes	Yes	Yes	Medium Risk
**Chin et al. 2017, Germany** [37]	No	No	Yes	Yes	Yes	Medium Risk
**Stokbro et al. 2016 USA** [38]	Yes	Yes	Yes	Yes	Yes	Low Risk
**Baan et al. 2016, Netherlands** [39]	Yes	No	Yes	Yes	No	Medium Risk
**Zhang et al. 2016, China** [40]	No	No	Yes	No	No	High Risk
**De Rio et al. 2014, Italy** [41]	Yes	No	Yes	Yes	No	Medium Risk
**Hsu et al. 2013, USA** [6]	No	Yes	Yes	Yes	Yes	Medium Risk
**Sun et al. 2013, Belgium** [7]	No	No	Yes	Yes	Yes	Medium Risk
**Zinser et al. 2013, Germany** [42]	No	No	Yes	Yes	Yes	Medium Risk
**Centenero and Hernández-Alfaro. 2012, Spain** [43]	No	No	Yes	Yes	Yes	Medium Risk

## Discussion

The use of computerized methods for diagnosis and treatment planning in orthodontics and orthognathic surgery has evolved substantially [42], which is confirmed by the 392 papers on this topic that have appeared in the major databases in the period (2007–2017).

Hsu and colleagues reported that computer-aided techniques enable the accurate correction of maxillary malformations with yaw deviation, alignment of proximal and distal segments and restoration of mandibular symmetry [6].

Lin and co-workers concluded that virtual orthognathic planning yields aesthetically favorable results, a high level of patient satisfaction, accurate translation of the treatment plan and thus making the operation itself easier and safer [20, 44].

The analyzed studies used both the CT and CBCT imaging modalities (two of them worked with both). Better identification of soft tissue and less image distortion where metallic elements are present are obvious advantages of CT over CBCT, while disadvantages include image quality, the supine position of the patient during the test (especially because of mandibular retrusion) and larger radiation doses [45–47]. Mandibular retrusion in the supine position during CT image capture was attenuated using central occlusal registry [6, 42]. The major disadvantage of CBCT is the occasional appearance of metal artifacts, but this is diminished by scanning the plaster casts [37, 40, 42], intraoral scanning of the dental arches [30, 37], scanning occlusion with reference points [6, 7] or by a triple scan procedure [39, 41].

Thus, the fusion of facial CT images and dental arch scans is important in computer-aided planning and it is more accurate when reference points are reproducible for both modalities [8].

### Evaluation of the accuracy of the virtual planning methods used in Orthognathic surgery

One of the most frequently used methods to evaluate the accuracy of virtual planning is the use of the mean error differences in superimposition between the virtual plan and the postoperative outcomes. Baan and colleagues used this technique to assess the degree of correspondence between the planned and performed positions. They also assessed the repeatability of the surgical procedure performed by different surgeons, and noticed that the discrepancy between the 3D planning and the postoperative results was the greatest regarding the vertical positioning of the maxilla and mandible, suggesting a less accurate intra-operative vertical control of virtual planning [39].

On the other hand, Franz and co-workers suggested that the use of the mean error as an only endpoint to measure the degree of accuracy can limit the generalizability of the studies. They also suggested that the confidence interval does not describe the real range of the method error but defines only the range of values that the mean error can assume from a statistical perspective [23].

Ho and colleagues calculated the accuracy of computer-aided orthognathic planning by evaluating the root-mean square difference (RMSD) of the 3D simulation and postsurgical CBCT images and found that the errors were acceptable, with RMSD (0.63 ± 0.25) mm for the maxilla and (0.85 ± 0.41) mm for the mandible [1].

De Riu and co-workers also suggested that the simple superimposition of the simulation and the cephalometric results is an unsatisfactory method, as it fails to consider the magnitude of the surgical manipulation leading to an error of a given magnitude. For instance, a slight positional error can be completely acceptable for large manipulations, but would be unacceptable when the manipulation takes place at a small scale and thus needs to be extremely precise [35].

The accuracy of the translation of the maxilla with computer-assisted planning for orthognathic surgery was < 1 mm in the study of Hsu and colleagues, indicating that this type of planning is accurate for the maxilla [6].

The Stokbro group found that the mean linear differences for the maxilla, mandible and the chin segment in all three planes were within 0.5 mm, while the mean precision, measured as the standard deviation, had the smallest deviation superoinferiorly, followed closely by mediolateral deviation, and finally the largest deviation was found anteroposteriorly [38].

De Riu and co-workers found that virtual surgical planning presented a high degree of accuracy for most of the parameters assessed, with an average error of 1.98 mm for linear measurements and 1.19° for angular measurements. At the same time, they observed significant differences between planned and achieved anterior facial height (*p* = 0.033). Without genioplasty, no significant difference was observed (U test; *p* = 0.45). The authors concluded that the problem was caused by the virtual model of the soft tissues, which made it difficult to manage the vertical dimension [35].

It has been also shown in the study of Baan and colleagues that the right /left translation has the lowest absolute mean difference between the 3D planning and the surgical results for both the maxilla and mandible (0.49 mm and 0.71 mm, respectively). Furthermore, they noticed that in 7 out of 10 cases, the maxilla was positioned more posteriorly than in the 3D plan, with an absolute mean difference of 1.41 mm. The same tendency was found in the sagittal position of the mandible,where in 8 out of 10 cases the mandible was positioned more posteriorly than planned with absolute mean difference of 1.17 mm [39]. Lee and colleagues suggested that the condylar position might have been changed during surgery by muscle tone and gravity as the patient was placed in the supine position, which affects the optimal condylar seating [48]. Stokbro et al. (2016) are of the same opinion about this issue.

The clinical analysis of Sun and colleagues, of the twenty three patients, using the OrthoGnathic Analyser, showed an adequate position of the maxilla and mandible in the left/right direction with a deviation of 0.32 mm and 0.75 mm, respectively. It was found that the maxilla had a lower RMSD (0.6 mm) than did the mandible (0.85 mm) [19].

Zhang et al. showed that the overall mean linear difference was (0.81 mm), and the overall mean angular difference was (0.95°) [40], which was an improvement as compared with their previous study, as a result of surgical experience, 3D printing technology, and improvement of the elasticity modulus of 3D-printed surgical templates [49].

On the other hand, Baan et al. observed that the accuracy of the pitch of the maxilla (2.72°) and the mandible (2.75°) showed the highest discrepancy between the 3D plans and the actual postoperative status. This variance could be the result of bone conflict between the pterygoid plate and the osteotimized maxilla [39]. Stokbro et al. came to similar conclusions [38].

### Comparison of the accuracy between classical and virtual planning methods

A lot of studies compared computer-assisted planning with classical planning and found favorable accuracy results in all bony segments for computer-aided planning [36, 41, 42, 50]. Ziesner and colleagues reported that the mandibular condyle maintained a central position in the temporomandibular joint, which did not occur when classic planning was used [42].

Hsu et al. compared the two types of interventions in the chin and found highly favorable accuracy results for computer-aided planning in this bone segment, with the largest difference recorded for translation in the sagittal plane (2.5 mm) and rotation pitch (3.68°). They explained these differences by the fact that classical planning does not use surgical splints; surgeons are guided by their experience, some internal reference points and the chin plate [6].

Ritto and colleagues reported on a similar level of precision in all evaluated regions when assessing the vertical positioning of the maxilla, but virtual surgical planning (VSP) was more accurate for the anteroposterior position of the maxilla. As for transverse positioning, conventional model surgery (CMS) yielded higher precision only for the upper midline position. However, there was no statistically significant difference between the groups, and the mean imprecision was also < 2 mm for all regions evaluated [36].

### Risk of Bias assessment

The papers included in this systematic review were classified as medium quality, since the risk of bias was considered medium in ten studies [1, 6, 7, 35–37, 39, 41–43], that is, the majority.

These studies [1, 7, 35–37, 42, 43] did not report on sample randomization and blinding. Baan et al. (2016) failed to report on blinding and follow-up.

## Conclusions

In conclusion, the results of this systematic review suggest that computer-aided planning is an accurate method for orthognathic surgery of the maxilla and the mandible.

We found that CBCT with intraoral scan of the dental cast is the most frequently used method for virtual orthognathic planning, and SimPlant (Materialise, Leuven, Belgium) and Dolphin (Dolphin Imaging, USA) are the most widely used software.

Despite its limitations, the calculation of the linear and angular differences between the virtual plan and the postoperative status is still the most frequently used method for accuracy assessment, and differences < 2 mm/° are considered acceptable.

## Supplementary Information

**Additional file 1: Table S1**. Virtual planning accuracy of the included studies.

## Data Availability

The datasets analyzed during the current study are available from the corresponding author on reasonable request.

## Abbreviations

CT: Computed tomography
CBCT: Cone-beam computed tomography
2D: Two-dimensional
3D: Three dimensional
OGS: Orthognathic surgery
MeSH: Medical subject heading
SD: Standard deviation
NA: No information provided by the authors
CMS: Conventional model surgery
VSP: Vitual surgical planning
M: Male
F: Female
FHP: Frankfort horizontal plane
CP: Coronal plane
MFP: Midfacial plane
OcPl: Occlusal plane
MdPl: Mandibular plane
N: Nasion point
ICC: Intra-class correlation coefficient
VS: Virtual splint
CS: Classic splint
NS: No splint
ANB: A relationship of maxilla and mandible
UAFH: Change of upper anterior facial height
LAFH: Change of lower anterior facial height
UAFH/LAFH: Change of vertical portion of facial height
RMSD: Root-mean square difference
