# Supplementary material for: Accuracy of virtual planning in orthognathic surgery: a systematic review
Source: Head Face Med. 2020 Dec 4;16:34. doi: 10.1186/s13005-020-00250-2 (PMC7716456; doi:10.1186/s13005-020-00250-2)
Supplement: Supplementary file 1 — Additional file 1: Table S1. Virtual planning accuracy of the included studies. [file 13005_2020_250_MOESM1_ESM.pdf]

Table S1. Virtual planning accuracy of the included studies.

Author and year	General<br>Mean , SD (variation)	Maxilla<br>Mean , SD (variation)	Mandible<br>Mean , SD (variation)	Chin<br>Mean , SD (variation)
**De Rio et al. 2017<br>Italy [35]**	**Angular measures (°)**<br>ANB : 0.2 ± 0.89 (P) 0.833<br>OcclTilt : 0.45 ± 0.95 (P) 0.6<br>**Linear measures (mm)**<br>AntFacH : 2.01 ± 1.45 (P) 0.033<br>PostFacH : 1.71 ± 1.67 (P) 0.442<br>DentMid : 0.58 ± 0.37 (P) 0.071<br>MxMdMid : -0.98± 0.39 (P) 0.235<br>B6–JawR : 0.6 ± 0.43 (P) 0.442<br>B6–JawL : 1.93 ± 1.47 (P) 0.263<br>DentJawMid : 0.06 ± 0.19 (P) 0.944	**Angular measures (°)**<br>A1-SN : -2.36 ± 1.99 , (P) 0.345<br>A1-FH : -5.01 ± 1.87 , (P) 0.064	**Angular measures (°)**<br>GonAng : 3.11 ± 1.60 (P) 0.093<br>**Linear measures (mm)**<br>Ramus H : -1.68 ± 1.66 , (P) 0.485	
**Ritto et al. 2017<br>Brazil [36]**	Vertical Mean (mm) : 1.44 ± 1.14<br>Anteroposterior Mean (mm) : 0.95 ± 1.04<br>Transverse Mean (mm) : 0.90 ± 0.87	**Vertical movements** (mm) **:**<br>11 (U1R) : 1.58 ± 1.25<br>21 (U1L) : 1.57 ± 1.32<br>16 (U6R) : 1.18 ± 0.61<br>26 (U6L) : 1.62 ± 1.57<br>13 (U3R) : 1.38 ± 0.96<br>23 (U3L) : 1.31 ± 1.01<br>**Transverse movements** (mm) **:**<br>Upper midline : 0.99 ± 0.87<br>16 (U6R) : 0.96 ± 0.89<br>26 (U6L) : 0.74 ± 0.89<br>**Anteroposterior movements** (mm) **:**<br>11 **(**U1R) : 1.01 ± 1.11<br>21 (U1L)) : 0.89 ± 1.00		
**Ho et al. 2017<br>Taiwan [1]**	**Angular measurments (°):**<br>Sagittal: Roll : 1.06 (0.23) 0.01<br>Vertical: Yaw : 1.88 (0.32) 0.00<br>Transversal : Pitch : 1.73 (0.4) 0.00			
**Chin et al. 2017<br>Germany [37]**	**Correction of occlusal plane :**<br>**Correction of U1 inclination :**<br>∠U1 axis-HP(°) : 4.31± 8.40 ,(p) 0.77<br>**Correction of anteroposterior position :**<br>∠ANB (°) : -0.67 ± 1.70 ,(p) 0,2<br>**Correction of anterior facial proportion**<br>UAFH (mm) : -1.22 ± 3,13 , (p) 0,37<br>LAFH (mm) : 2,74 ± 2,49, ,(p) 0,87<br>UAFH/ LAFH (%):-4.50 %± 5.47 %,(p) 0,12	**Correction of midline:**<br>**Correction of maxillary segment**<br>∠ MxCP - MSP : 0.54± 4.61,(p) 0,58<br>**Correction of occlusal plane**<br>Correction of maxillary OP<br>∠ MxOP – HP : -0.27 ± 5,1,(p) 0,55<br>**Correction of anteroposterior position**<br>AP position of maxilla<br>A-CP (mm) : -2.38± 2,06,(p) 0,88	**Correction of midline :**<br>**Correction of mandible segment**<br>∠ MdCP -MSP : 1.18 ± 2,63 ,(p) 0,74<br>**Correction of occlusal plane**<br>Correction of mandibular OP<br>∠ MdOP – HP: -0.46 ± 3,55 ,(p) 0,40<br>**Correction of anteroposterior position**<br>AP position of mandible<br>Pog-CP (mm) : -0.48 ± 6,05 ,(p) 0,37	

**Stokbro et al. 2016<br>USA [38]**	**Segmentation of the maxilla (mm) :**<br>**Segmented maxilla :**<br>Mediolateral : -0.15 ± 0.90 (p) 0.567<br>Anteroposterior : -0.87 ± 2.85 (p) 0.290<br>Superoinferior : -0.10 ± 1.24 (p) 0.779<br>**Non-segmented maxilla :**<br>Mediolateral : -0.43 ± 1.72 (p) 0.319<br>Anteroposterior : -0.02 ± 1.96 (p) 0.962<br>Superoinferior : 0.00 ± 1.50 (p) 0.992<br>**Influence of genioplasty (mm) :**<br>**With genioplasty:**<br>Mediolateral : -0.60 ± 1.70 (p) 0.345<br>Anteroposterior : 0.20 ± 4.50 (p) 0.917<br>Superoinferior : -1.21 ± 1.82 (p) 0.249<br>**Without genioplasty :**<br>Mediolateral : -0.01 ± 1.97 (p) 0.647<br>Anteroposterior : -0.28 ± 3.28 (p) 0.879<br>Superoinferior : -0.14 ±0.52 (p) 0.317	**Linear differences(mm) :**<br>Mediolateral : -0.31 ±1.41 (p) 0.284<br>Anteroposterior : -0.39 ± 2.38 (p)0.237<br>Superoinferior : -0.05 ± 1.37 (p) 0.644<br><br>**Rotational differences(°) :**<br>Roll : - 0.03 ± 0.53 (p) 0.820<br>Pitch :-0.54 ± 0.91 (p) 0.006<br>Yaw :0.17 ± 0.48 (p) 0.072<br><br>**Transverse expansion(mm) :**<br>**Segmented** : -1.41 ±1.01 (p) 0.002<br>**Non-segmented** : 0.00 ± 0.05 (p) 0.549	**Linear differences (mm):**<br>Mediolateral : -0.14 ±1.40 (p) 0.910<br>Anteroposterior : 0.28 ± 2.08 (p) 0.658<br>Superoinferior : 0.13 ±0.73 (p) 0.614<br><br>**Rotational differences (°):**<br>Roll : -0.04 ± 0.44 (p) 0.629<br>Pitch : - 0.14 ± 0.75 (p) 0.504<br>Yaw : 0.13± 0.40 (p) 0.046	**Menton (Linear differences(mm):**<br>Mediolateral :<br>-0.16 ±, 1.89 (p) 0.951<br>Anteroposterior :<br>-0.16 ± 3.52 (p) 0.977<br>Superoinferior:<br>-0.41 ± 1.07 (p) 0.110
**Baan et al. 2016<br>Netherlands [39]**		**Linear differences(mm) :**<br>Translation AP : 1.41<br>Translation LR : 0.49<br>Translation UD : 1.85<br>**Angular differences(°) :**<br>Pitch : 2.72<br>Roll : 1.04<br>Yaw : 0.97	**Linear differences(mm) :**<br>Translation AP : 1.17<br>Translation LR : 0.71<br>Translation UD : 1.32<br>**Angular differences(°) :**<br>Pitch : 2.75<br>Roll : 0,84<br>Yaw : 1,13	
**Zhang et al. 2016<br>China [40]**	**Symmetry planes**<br><br>**Angular difference relative to FHP (°)**<br>Occlusal plane 1.1 ± 0.6<br>Palatal plane 0.8 ± 0.3<br>Mandibular plane 1.3 ± 0.6<br><br>**Angular difference relative to midfacial plane(°)**<br>Occlusal plane : 0.9 ± 0.4<br>Palatal plane : 0.6 ± 0.2<br>Mandibular plane : 1.0 ± 0.6	**Differences of the distances (mm)**<br>**Difference of the distance to FHP :**<br>UI : 0.7±0.3<br>U6 (R) : 0.8 ± 0.4<br>U6 (L) : 0.7 ± 0.3<br>**Difference of the distance to midfacial plane**<br>UI :0.4 ± 0.1<br>U6 (R) : 0.6 ± 0.2<br>U6 (L) :0.5 ± 0.2<br>**Difference of the distance to coronal plane**<br>UI : 0.8 ± 0.4<br>U6(R) :0.9 ± 0.3<br>U6(L) : 1.0 ± 0.5	**Differences of the distances (mm)**<br>**Difference of the distance to FHP :**<br>LI : 1.1 ± 0.5<br>L6 (R) : 1.0 ±0.4<br>L6 (L) : 1.2 ± 0.5<br>**Difference of the distance to midfacial plane**<br>LI : 0.5 ± 0.3<br>L6(R) : 0.7 ± 0.3<br>L6(L) :0.6 ± 0.3<br>**Difference of the distance to coronal plane**<br>LI : 1.0 ± 0.4<br>L6(R) : 1.1± 0.6<br>L6 (L) :1.0 ±0.5	
**De Rio et al. 2014<br>Italy [41]**		**Bone tissue**<br>Vertical: 92.59% (VS)<br>Transversal: < 88.1% (VS)	**Bone tissue**<br>Vertical: 92.59% (VS) : 58.5% (CS)<br>Transversal: <88.17% (VS) : <50.82% (CS)	**Bone tissue**<br>Transversal: 85.77%<br>Transversal: 76.67%
**Hsu et al. 2013<br><br>USA [6]**	NA	Sagittal (mm): 1 (-0.7 to 1.6)<br>Roll**(°):** 0.9 (-1.8 to 1.8)	Sagittal(mm): 1.1 (-0.9 to 1.5)<br>Roll**(°):** 1 (-2 to 1.8)	**Sagittal(mm)**:<br>1 (-2.1to2) (VS)

		**Vertical (mm):** 0.6 (- 0.8 to 0.9)<br>Yaw **(°):** 1.3 (-2.7 to 2.3)<br><br>**Transversal(mm):** 0.8 (-1.7 to1.4)<br>Pitch**(°):** 1.5 (-2.3 to 3.4)	**Vertical(mm):** 0.6 (-0.8 to 0.7)<br>Yaw**(°):** 1.7 (-3.3 to 3.3)<br><br>**Transversal(mm):** 0.8(-1.4 to 1)<br>Pitch**(°):** 1.8 (-3.7 to 3.6)	3.5 (-6.2to7.8) (NS)<br>**Roll(°):**<br>1.88 (-4.8 to 3.78) (VS)<br>3.8 (-5.88 to 6.38) (NS)<br>**Vertical(mm)**:<br>0.6(-1.4to1) (VS)<br>2.5(-5.3 to 4.6) (NS)<br>**Yaw(°):**<br>1.98(-48 to 4.18) (VS)<br>3.98 (-7.18 to 8.48) (NS)<br>**Transversal(mm)**:<br>0.8(-1.7 to 1.8) (VS)<br>1.7 (-2.9 to 3.9)(NS)<br>**Pitch(°):**<br>2.28 (-4.18 to 4.98) (VS)<br>5.88 (-9.48 to12.98)(NS)
**Sun et al. 2013<br>Belgium [7]**	NA	**Linear differences**(mm) **:**<br>**Sagittal** : 0.5 ± 0.22<br>**Vertical** : 0.57± 0.35<br>**Transversal** : 0.38 ±0.35	NA	NA
**Zinser et al. 2013<br>Germany [42]**	NA	**Bone tissue**<br>Sagittal (mm): <0.14<br>Vertical (mm): <0.23<br>Transversal (mm): <0.04<br>MxPl: 0.358° (FHP)—0.038° (MFP)<br>OcPl: 0.028° (FHP)—0.038° (MFP)	**Bone tissue**<br><br>Sagittal (mm): < 0.17<br>Vertical(mm): < 0.33<br>Transversal(mm): < 0.17<br>MdPl: 0.588 (FHP)—0.618 (MFP)	NA
**Centenero and Hernández-Alfaro . 2012<br>Spain [43]**	**Bone tissue**<br>0.722±0.246 (0.350–0.964) ICC<br>**Angles**: 0.655±0.249 (0.350–0.910) ICC<br>**Lines**: 0.922±0.059 (0.880– 0.964) ICC	**OcPl**: 0.375(-0.178 to0.739)ICC (FHP)	**MdPl**: 0.608(0.162to0.849) ICC (FHP)	NA

SD: standard deviation;NA,information not provided by theauthors; ICC, intra-class correlation coefficient ;OcPl, occlusal plane; FHP, Frankfort horizontal plane; MdPl, mandibular plane; VS, virtual splint ;NS , no splint; CS, classic splint; MxPl, maxillary plane; MFP, midfacial plane, ANB, A relationship of maxilla and mandible ; UAFH , Change of upper anterior facial height; LAFH , Change of lower anterior facial height ; UAFH/LAFH , Change of vertical portion of facial height .
